# Supplementary material for: Precision arbovirus serology with a pan-arbovirus peptidome
Source: Nat Commun. 2024 Jul 11;15:5833. doi: 10.1038/s41467-024-49461-0 (PMC11239951; doi:10.1038/s41467-024-49461-0)
Supplement: Supplementary file 1 — Supplementary Information [file 41467_2024_49461_MOESM1_ESM.pdf]

Supplementary information for

Precision Arbovirus Serology with a Pan-Arbovirus Peptidome

Author list

William R. Morgenlander[1,2], Wan Ni Chia[3], Beatriz Parra[4], Daniel R. Monaco[5], Izabela Ragan[6], Carlos A. Pardo[1,7], Richard Bowen[6], Diana Zhong[8], Douglas E. Norris[9], Ingo Ruczinski[10], Anna Durbin[11], Lin-Fa Wang[3], H. Benjamin Larman[1,2]*, Matthew Robinson[8]*

Affiliations

[1] Department of Pathology, Johns Hopkins University School of Medicine, Baltimore, MD, USA
[2] Institute for Cell Engineering, Johns Hopkins University School of Medicine, Baltimore, MD, USA
[3] Program in Emerging Infectious Diseases Duke-NUS Medical School, Singapore
[4] Department of Microbiology, Universidad del Valle, Cali, Colombia
[5] ImmuneID, Waltham, MA, USA
[6] Department of Biomedical Sciences, Colorado State University College of Veterinary and Biomedical Sciences, Fort Collins, CO, USA
[7] Department of Neurology, Johns Hopkins University School of Medicine, Baltimore, MD, USA
[8] Department of Medicine, Johns Hopkins University School of Medicine, Baltimore, MD, USA
[9] Department of Molecular Microbiology and Immunology, Johns Hopkins University Bloomberg School of Public Health, Baltimore, MD, USA
[10] Department of Biostatistics, Johns Hopkins University Bloomberg School of Public Health, Baltimore, MD, USA
[11] Department of International Health, Johns Hopkins University Bloomberg School of Public Health, Baltimore, MD, USA

* denotes co-corresponding authors

Address correspondence to:
H. Benjamin Larman, PhD - hlarman1@jhmi.edu
Matthew Robinson, MD - mrobin85@jhmi.edu

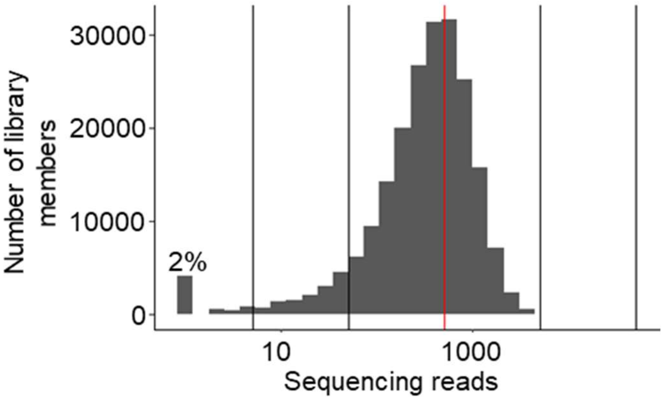

Supplementary Figure 1

After the ArboScan phage library was cloned, the peptide coding region of was sequenced to establish cloning efficacy. The number of sequencing reads attributed to each library member was tabulated and displayed as a histogram. 98% of library members were successfully cloned, as indicated by >0 reads, and 97% of library members are represented within 100-fold of the mean.

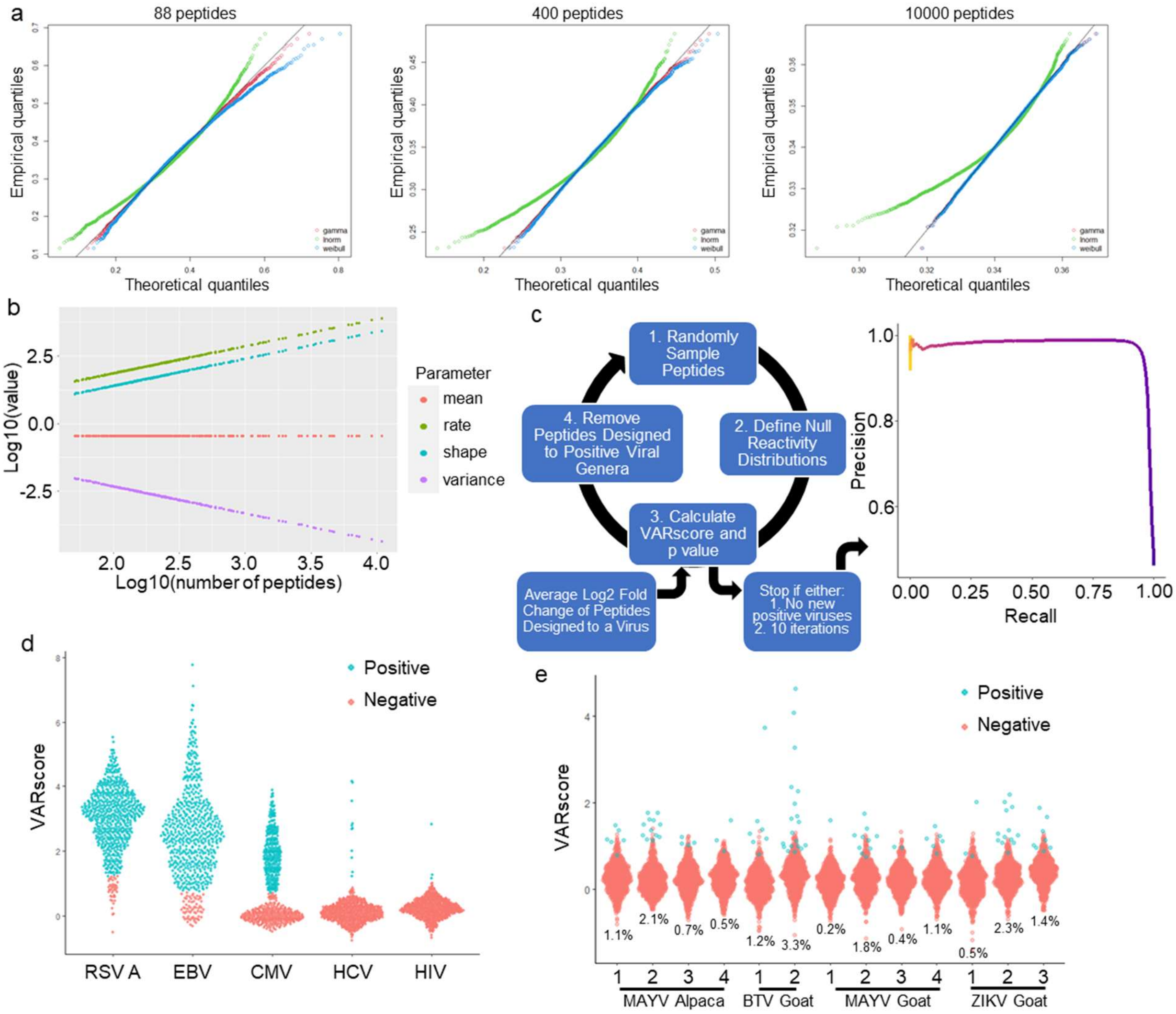

Supplementary Figure 2

a) QQ plots were used to evaluate Gamma, log normal, and Weibull distributions fit to random distributions made by selecting 88, 400, and 10000 random ArboScan peptides 1000 times. Gamma distributions were empirically determined to best fit the random distributions, and were used in the VARscore algorithm. b) Best fit gamma distribution parameters were compared to the number of peptides selected in generating random distributions. Parameters vary regularly with the number of peptides selected, so in the VARscore algorithm parameters for intermediate numbers of peptides were interpolated using a linear model. c) Schematic of the VARscore algorithm. Average reactivity to peptides designed to a virus are compared to randomly selected peptides from seronegative viruses to define the magnitude of antibody responses to individual viruses. Precision recall curve when applied to the VRC cohort VARscores for viruses in table S3. d) VARscores for each individual in the VRC cohort (n = 615) for respiratory syncytial virus A (RSV A), Epstein-Barr virus (EBV), cytomegalovirus (CMV), hepatitis C virus (HCV), and human immunodeficiency virus 1 (HIV). A VARscore was considered positive if it was greater than the optimal cutoff determined via the ROC analysis shown in Fig 1 and had an associated p value less than 0.0001 for a virus. e) All VARscores from viruses in ArboScan and percentage of viruses positive for pre-challenge samples from the veterinary cohort as well as from the blue tongue virus (BTV) Goats that lacked neutralizing BTV antibodies. The same positivity criteria were used as in panel d. MAYV - Mayaro virus, ZIKV - Zika virus

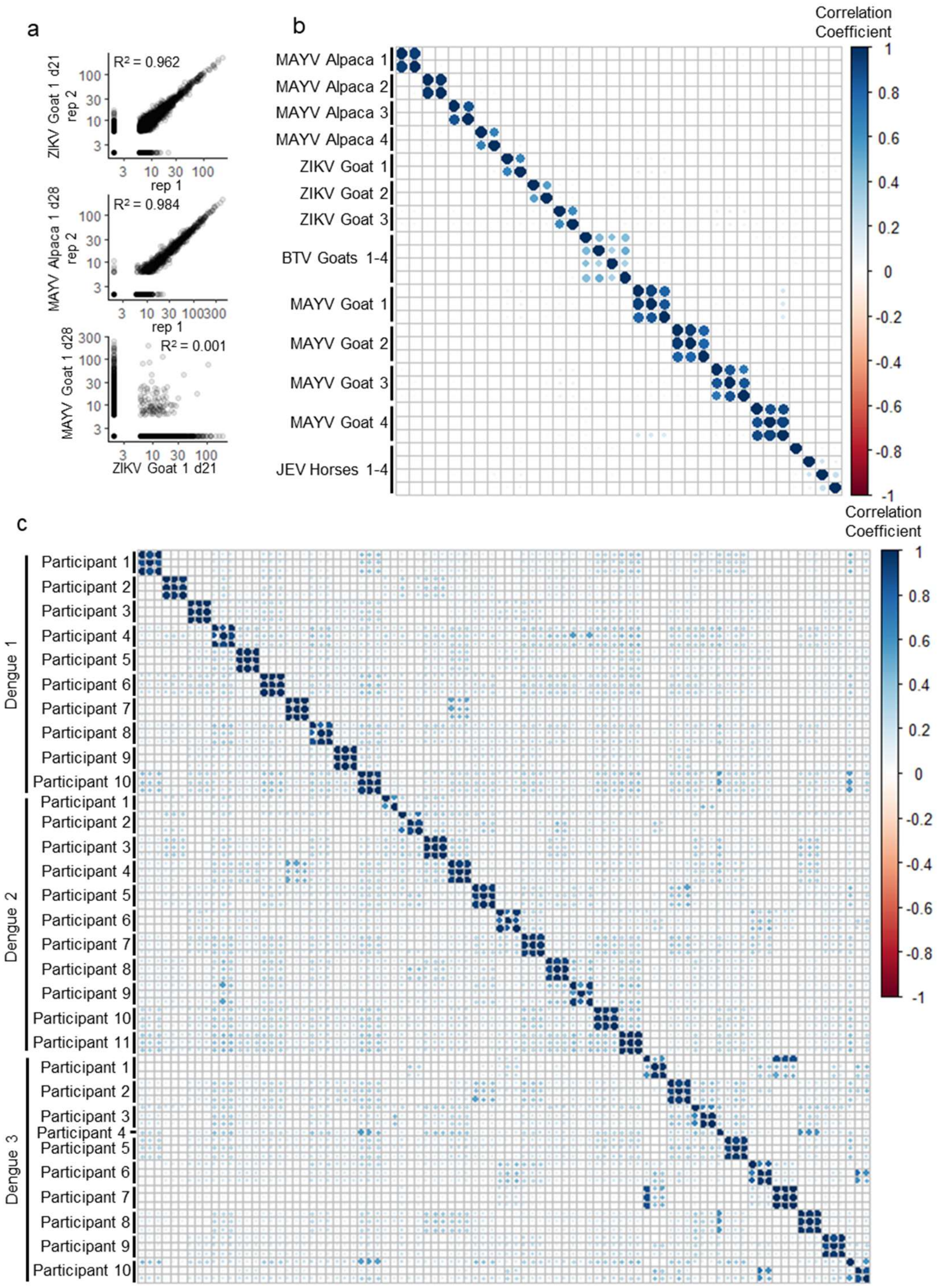

Supplementary Figure 3

a) one goat sample and one alpaca sample were profiled in duplicate, and replicate runs were compared (Pearson correlation, Goat R^2 = 0.962, Alpaca R^2 = 0.984). Each point represents one ArboScan peptide. Two unrelated goat samples are included for comparison (Pearson correlation, R^2 = 0.001). b-c) Correlation plots were made for ArboScan antibody profiles for the veterinary cohort (b) and the dengue challenge cohort (c). Antibody profiles from different timepoints for the same individual were highly correlated.

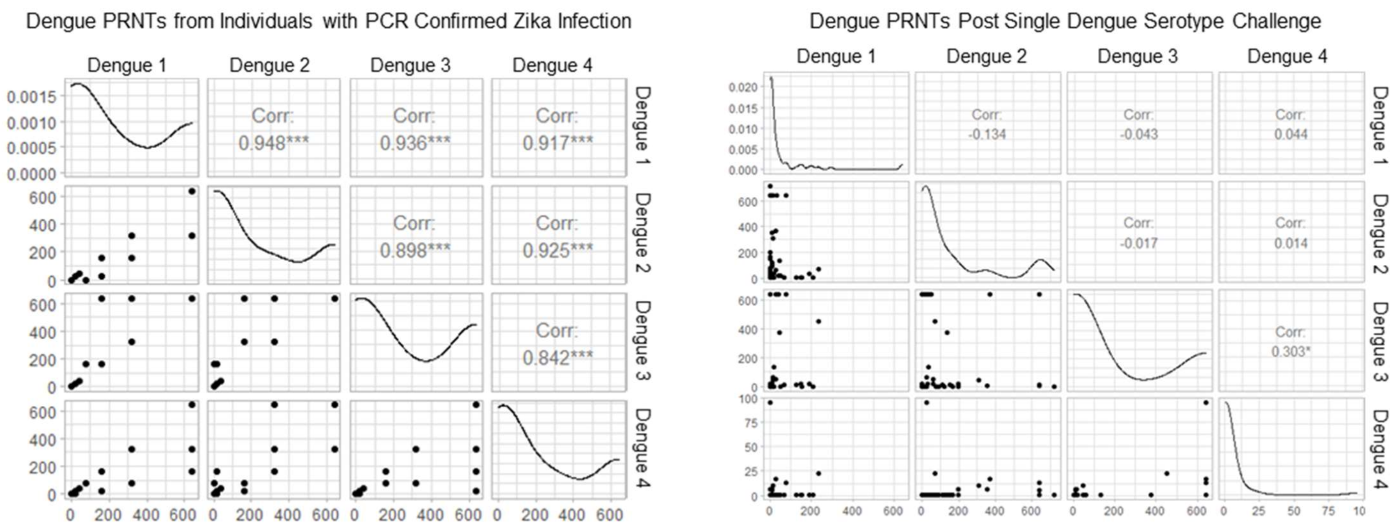

Supplementary Figure 4

Pairs plots were made for dengue plaque reducing neutralizing titer (PRNT) for the Colombia cohort (left) and the dengue challenge cohort (right). Neutralization of each dengue serotype correlated with neutralization of all other serotypes in the Colombia Zika cohort by pearson correlation. Neutralization of each dengue serotype was not correlated with neutralization of any other serotype in the dengue challenge cohort with the exception of the weak correlation between dengue-3 and dengue-4. * denotes $p < 0.05$, *** denotes $p < 0.001$

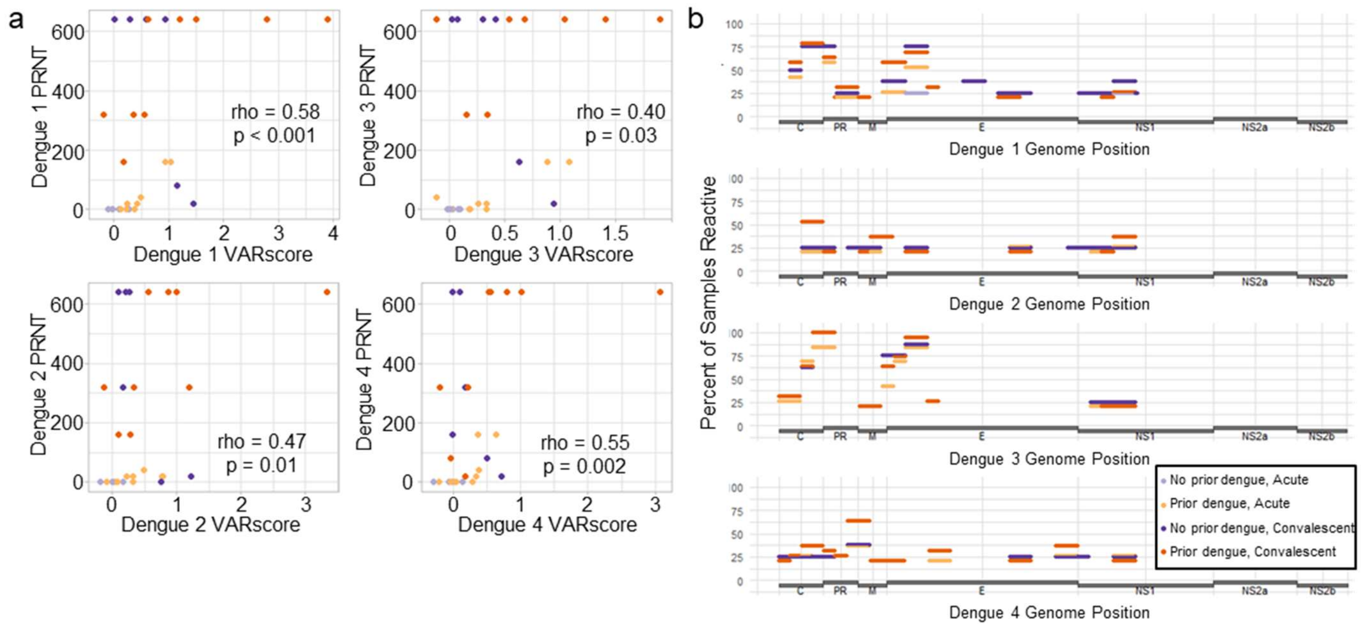

Supplementary Figure 5

a) For all samples in the Colombia cohort, each dengue serotype VARscore was compared to the corresponding dengue serotype plaque reducing neutralizing titer (PRNT). Each serotype VARscore correlates with the corresponding serotype PRNT (Spearman correlation, dengue 1 $p < 0.001$, dengue 2 $p = 0.01$, dengue 3 $p = 0.03$, dengue 4 $p = 0.002$). b) Prevalence of each dengue epitope reactivity was calculated for each sample group in the Colombia Zika cohort. Following Zika infection, individuals with ($n = 19$) and without ($n = 8$) pre-existing Flavivirus antibodies often produced antibodies that bound multiple dengue virus epitopes. Targets of dengue-reactive antibodies induced by Zika infection included structural proteins and NS1, but no antibodies targeting dengue NS2b were detected.

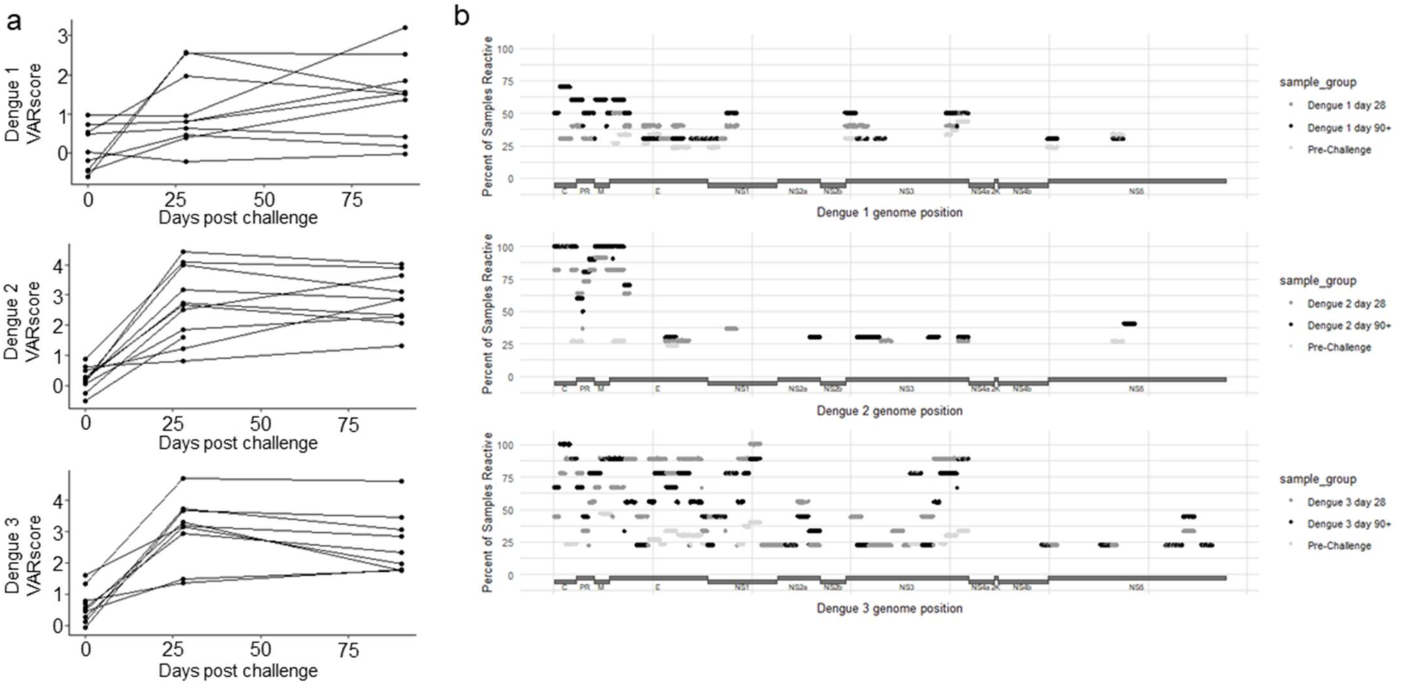

Supplementary Figure 6

a) Individuals in the dengue challenge cohort were exposed to dengue 1, 2, or 3, and VARscore for the challenge dengue serotype were evaluated longitudinally (Dengue 1: day 0 n=10, day 28 n =10, day 90+ n = 10; Dengue 2: day 0 n = 11, day 28 n = 11, day 90 n = 10; Dengue 3: day 0 n = 10, day 28 n = 9, day 90 n = 9). b) Prevalence of epitope reactivities to the relevant challenge dengue strain were calculated for individuals challenged with dengue 1, 2, and 3 post challenge. Many individuals produced delayed antibody responses to capsid (C) and membrane glycoprotein precursor (Pr). Abbreviations: membrane (M), envelope (E), non-structural protein (NS)

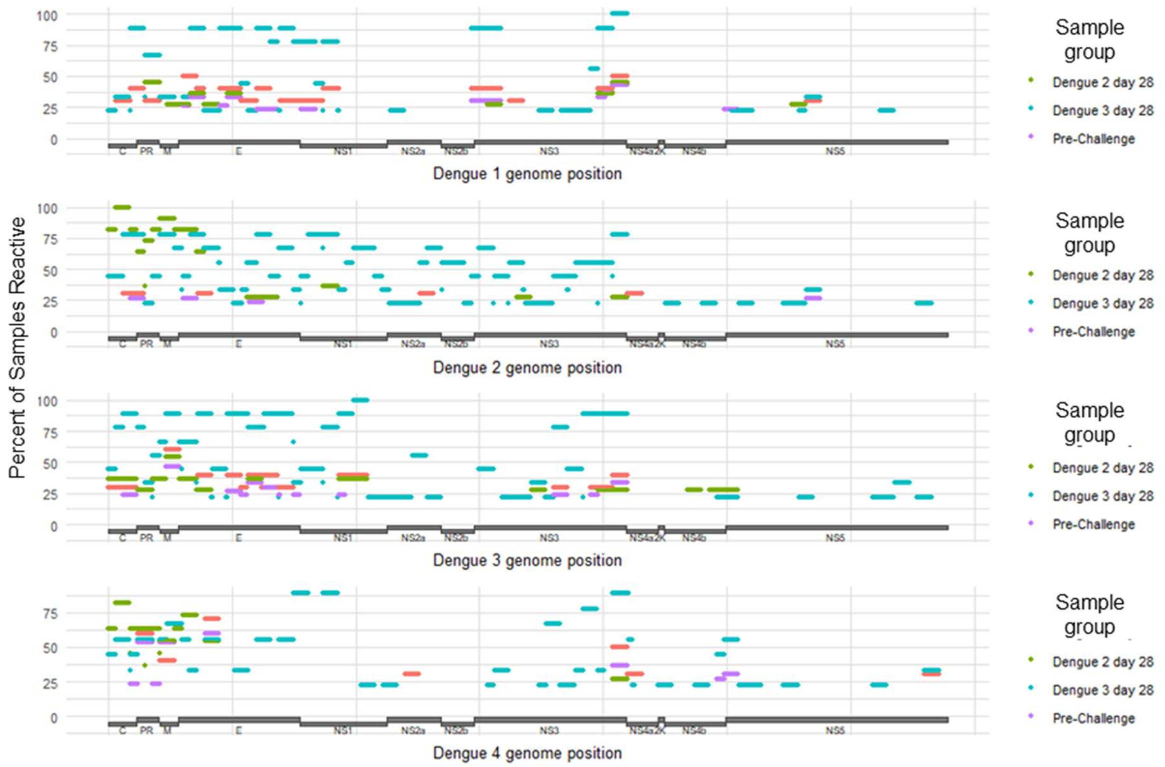

Supplementary Figure 7

Prevalence of antibody reactivity to dengue epitopes was calculated before and after challenge with dengue 1, 2, and 3. Preexisting antibodies most frequently target several regions: the center of envelope (E) (dengue-1 poly-protein amino acids 482-623, dengue-1 E amino acids 201-422), the C terminus of non-structural protein 3 (NS) (dengue-1 poly-protein amino acids 2025-2120, NS3 amino acids 549-618), and the center of NS5 (dengue-1 polyprotein amino acids 2820-2878, NS5 amino acids 326-384). Dengue-1 challenge elicited marginal increases in antibodies reactive to the N terminus of dengue-1 E, as well as to dengue-1 NS1. Following challenge with dengue-2, >75% of individuals developed antibodies reactive to the N terminus of dengue-2 E, capsid (C), membrane glycoprotein precursor (Pr), and membrane (M), as well as antibodies reactive to homologous peptides from other dengue viruses. Additionally, a subset of patients developed antibodies to NS1. Dengue-3 challenge resulted in the most robust antibody responses. 89% of patients developed a cross-reactive antibody response to the N terminus of envelope, 100% of patients developed antibodies to cross-reactive antibodies to NS1 and the C terminus of NS3.

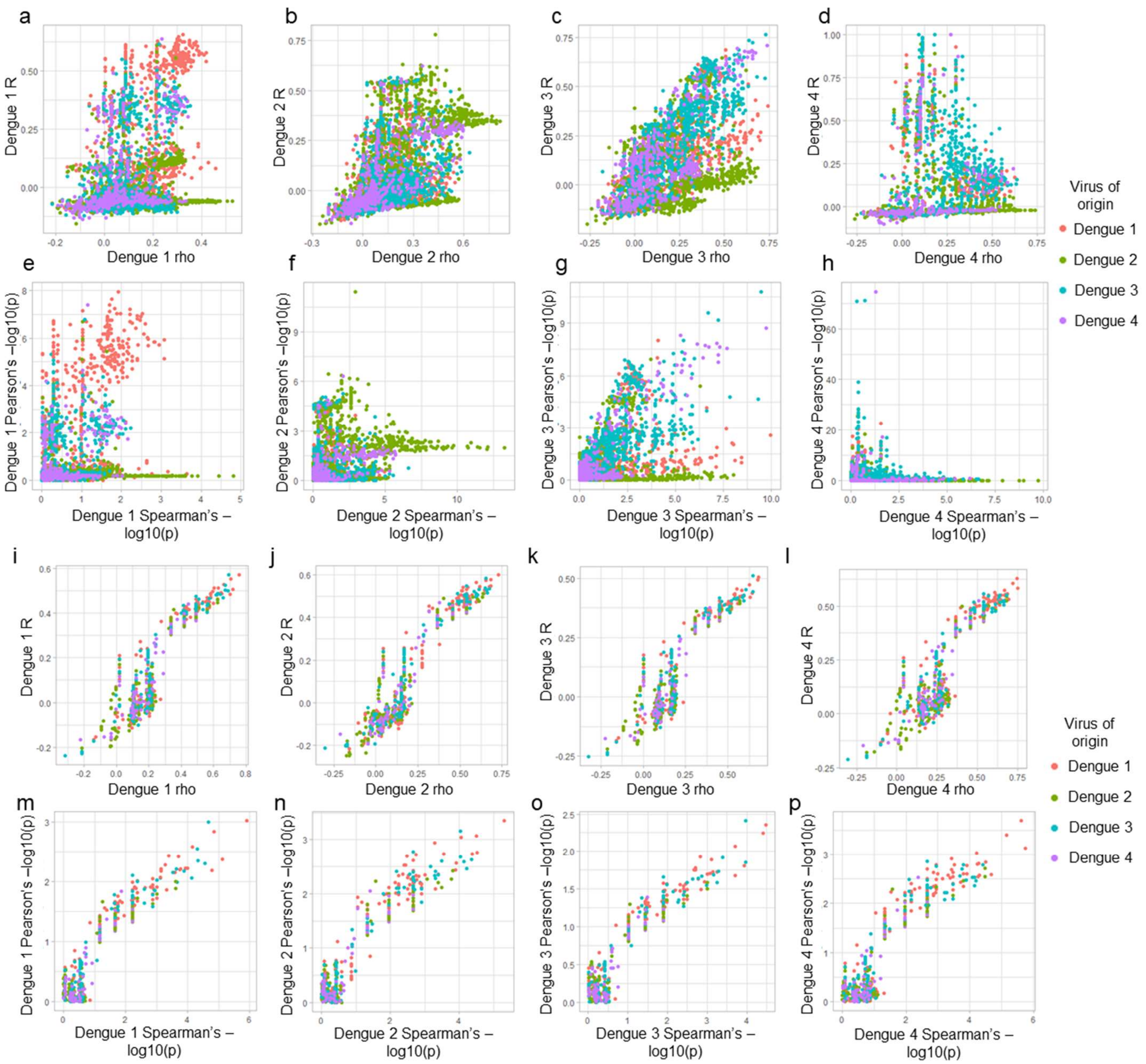

Supplementary Figure 8

a-h) Reactivity to each dengue peptide in ArboScan was evaluate for correlation with neutralization of each dengue serotype in the dengue challenge cohort. a-d) Pearson's R and Spearman's rho for the correlation between each dengue peptide in ArboScan and dengue 1 PRNT (a), dengue 2 PRNT (b), dengue 3 PRNT (c), and dengue 4 PRNT (d). e-h) p values from correlations in a-d. i-p) Reactivity to each dengue peptide in ArboScan was evaluate for correlation with neutralization of each dengue serotype in the Colombia cohort. i-l) Pearson's R and Spearman's rho for the correlation between each dengue peptide in ArboScan and dengue 1 PRNT (i), dengue 2 PRNT (j), dengue 3 PRNT (k), and dengue 4 PRNT (l). m-p) p values from correlations in i-l.

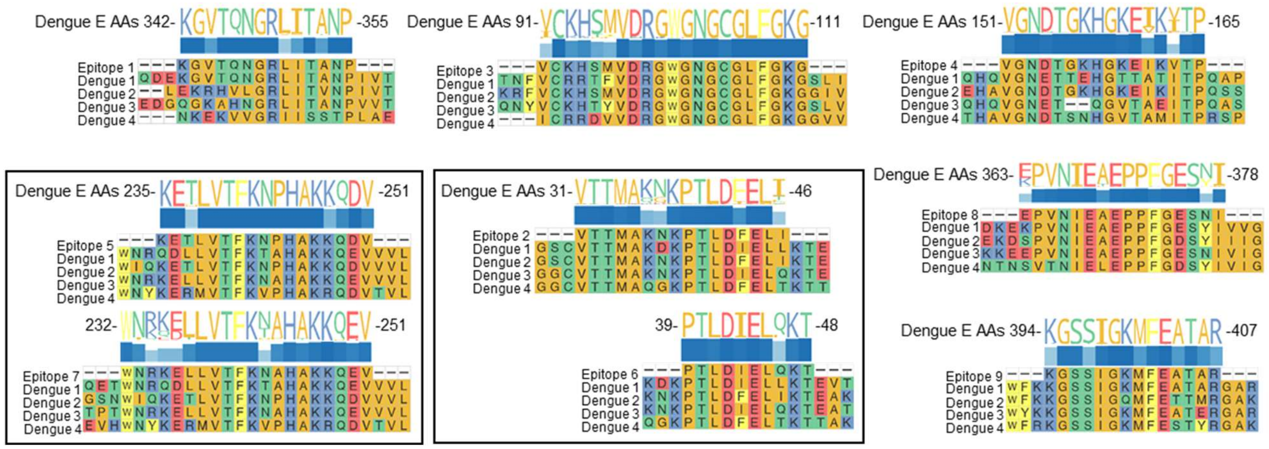

Supplementary Figure 9

Minimal epitopes were defined that may explain the correlations between dengue envelope peptides and dengue neutralization (Fig 5). Epitope icons made from overlapping peptides that correlate with neutralization are shown and compared to dengue 1-4 reference sequences (see methods). Epitopes 5 and 7 overlap, as do epitopes 2 and 6, so these are displayed aligned to each other. MSAs show the consensus sequence for the epitope aligned to dengue 1-4 envelope reference sequences. Dengue envelope amino acid number for epitope 1: AAs 342-355, epitope 2: AAs 31-46, epitope 3: AAs 91-111, epitope 4: AAs 151-165, epitope 5: AAs 235-251, epitope 6: AAs 39-48, epitope 7: AAs 232-251, epitope 8: AAs 363-378, and epitope 9: AAs 394-407

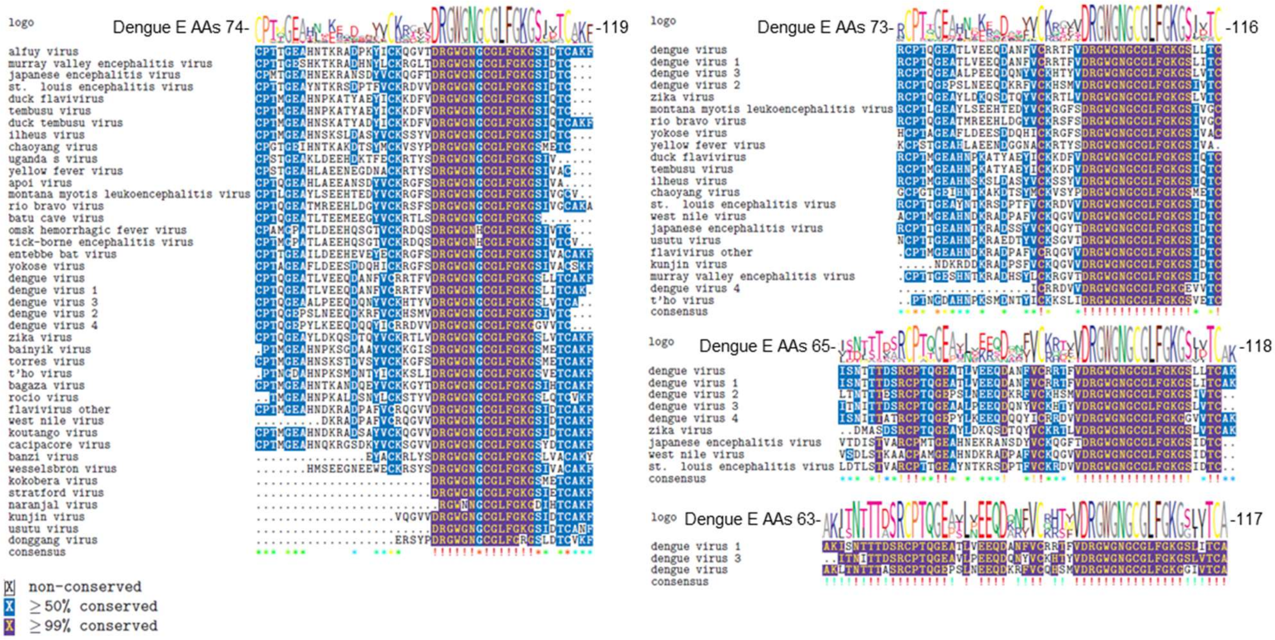

Supplementary Figure 10

Fusion loop epitope icons and MSAs for four example sera demonstrating differential degrees of cross-reactivity. Icons were made using the maximally reactive peptide overlapping fusion loop from each reactive virus for that individual. Icon width was selected to include all residues with >50% conservation across each reactive virus.

Viruses only known to infect arthropods	
Genus	Strain
rhabdovirus	Shuangau bedbug virus
rhabdovirus	Yongjia tick virus 2
phlebovirus	Yongjia tick virus 1
flavivirus	Mosquito flavivirus
NA	Tacheng tick virus
peribunyavirus	Huangpi tick virus 1
phlebovirus	Huangpi tick virus 2
rhabdovirus	Huangpi tick virus 3
NA	Wuhan mosquito virus
rhabdovirus	Wuhan tick virus 2
peribunyavirus	wenzhou tick virus
Highly represented viruses with low human health priority	
Genus	Strain
orthobunyavirus	Schmallenberg virus
rhabdovirus	Bovine ephemeral fever virus
orbivirus	Equine encephalosis virus
orbivirus	epizootic hemorrhagic fever virus
orbivirus	african horse sickness virus
orbivirus	bluetongue virus
rhabdovirus	vesicular stomatitis virus

Supplementary Table 1

Species & peptides by arbovirus genus		
Genus	Species	Peptides
Alphavirus	39	19280
Coltivirus	2	1028
Control	5	5014
Flavivirus	114	80766
Other	2	891
Nyavirus	3	363
Orbivirus	62	22572
Orthobunyavirus	135	22380
Orthonairovirus	42	16564
Phlebovirus	108	17856
Quaranjavirus	3	277
Rhabdoviridae genus	129	17999
Thogotovirus	7	967
Peribunyaviridae genus	45	5019
Total	696	210,976

Supplementary Table 2

Viruses expected to be frequently targeted	
Genus	Species
Enterovirus	35 strains
Lymphocryptovirus	Epstein-Barr Virus
Cytomegalovirus	Human Cytomegalovirus
Orthopneumovirus	3 strains of Human Respiratory Syncytial Virus
**Viruses expected to be rarely or never targeted**	
Genus	Species
Ebolavirus	5 strains of ebolavirus
Coltivirus	Colorado tick fever virus
Marburgvirus	Lake Victoria marburgvirus
Flavivirus	15 species
Gammaretrovirus	2 species
Thogotovirus	Dhori virus
Hepacivirus	19 strains of Hepatitis C virus
Yatapoxvirus	3 species

Supplementary Table 3
