# Supplementary material for: Precision arbovirus serology with a pan-arbovirus peptidome
Source: Nat Commun. 2024 Jul 11;15:5833. doi: 10.1038/s41467-024-49461-0 (PMC11239951; doi:10.1038/s41467-024-49461-0)
Supplement: Supplementary file 3 — Reporting Summary [file 41467_2024_49461_MOESM3_ESM.pdf]

nature portfolio

Corresponding author(s): H. Benjamin Larman and Matthew Robinson

Last updated by author(s): 12/18/2023

Reporting Summary

Nature Portfolio wishes to improve the reproducibility of the work that we publish. This form provides structure for consistency and transparency in reporting. For further information on Nature Portfolio policies, see our Editorial Policies and the Editorial Policy Checklist.

Statistics

For all statistical analyses, confirm that the following items are present in the figure legend, table legend, main text, or Methods section.

n/a	Confirmed	
☐	☒	The exact sample size (*n*) for each experimental group/condition, given as a discrete number and unit of measurement
☐	☒	A statement on whether measurements were taken from distinct samples or whether the same sample was measured repeatedly
☐	☒	The statistical test(s) used AND whether they are one- or two-sided<br>*Only common tests should be described solely by name; describe more complex techniques in the Methods section.*
☒	☐	A description of all covariates tested
☐	☒	A description of any assumptions or corrections, such as tests of normality and adjustment for multiple comparisons
☐	☒	A full description of the statistical parameters including central tendency (e.g. means) or other basic estimates (e.g. regression coefficient) AND variation (e.g. standard deviation) or associated estimates of uncertainty (e.g. confidence intervals)
☐	☒	For null hypothesis testing, the test statistic (e.g. *F*, *t*, *r*) with confidence intervals, effect sizes, degrees of freedom and *P* value noted<br>*Give P values as exact values whenever suitable.*
☒	☐	For Bayesian analysis, information on the choice of priors and Markov chain Monte Carlo settings
☒	☐	For hierarchical and complex designs, identification of the appropriate level for tests and full reporting of outcomes
☐	☒	Estimates of effect sizes (e.g. Cohen's *d*, Pearson's *r*), indicating how they were calculated

Our web collection on statistics for biologists contains articles on many of the points above.

Software and code

Policy information about availability of computer code

Data collection	Sequencing data was collected using Illumina Real-Time Analysis software on the sequencing instrument. We utilized exact matching implemented in R v 4.2.2 to map sequencing reads to PhIP-Seq library members producing counts data.
Data analysis	Data analysis was performed in R v 4.2.2 unless otherwise stated. Enrichment of antibody reactivities was determined via the edgeR package (v.3.32.0). Packages used for analysis include Biostrings (v 2.66.0) and ARscore (v 0.2.0). The ARscore package was developed as part of this manuscript and is available at https://github.com/wmorgen1/ARscore. Plots were generated with the ggplot2 package (v 3.4.0), ggtree (v 3.4.4), circlize (v 0.4.15), msa (v 1.28.0), and pheatmap (v 1.0.12). Phylogenies were created with MEGA X.

For manuscripts utilizing custom algorithms or software that are central to the research but not yet described in published literature, software must be made available to editors and reviewers. We strongly encourage code deposition in a community repository (e.g. GitHub). See the Nature Portfolio guidelines for submitting code & software for further information.

Data

Policy information about availability of data

All manuscripts must include a data availability statement. This statement should provide the following information, where applicable:

- Accession codes, unique identifiers, or web links for publicly available datasets
- A description of any restrictions on data availability
- For clinical datasets or third party data, please ensure that the statement adheres to our policy

Raw and processed data generated for this study are freely available from authors on request.

Research involving human participants, their data, or biological material

Policy information about studies with human participants or human data. See also policy information about sex, gender (identity/presentation), and sexual orientation and race, ethnicity and racism.

Reporting on sex and gender	Sex and gender were not considered in this study. All clinical specimens were deidentified and these data were not provided to the study team.
Reporting on race, ethnicity, or other socially relevant groupings	Social categorizations were not considered in this study. All clinical specimens were deidentified and these data were not provided to the study team.
Population characteristics	Age and genotype information were not included as covariates in this study. Additional serologic information in the form of clinical ELISAs and Neutralizing titers were utilized as described in the methods.
Recruitment	Cohort 2: participants referred to the Universidad del Valle Virology Laboratory, Cali, Clombia and had PCR confirmed Zika infection were recruited.<br>Cohort 3: Healthy volunteers without existing evidence of flavivirus exposure were recruited to participate in dengue vaccine trials
Ethics oversight	The cohort 2 study protocol was approved by the institutional review boards at Universidad del Valle (protocol 034-016, IRB Approval # 006-016) and the Johns Hopkins University School of Medicine (IRB IRB00093149).<br>The cohort 3 study protocols were approved by IRBs at the University of Vermont and Johns Hopkins Bloomberg School of Public Health.<br>All samples were deidentified and this study team was not given access to clinical data.

Note that full information on the approval of the study protocol must also be provided in the manuscript.

Field-specific reporting

Please select the one below that is the best fit for your research. If you are not sure, read the appropriate sections before making your selection.

- [x] Life sciences
- [ ] Behavioural & social sciences
- [ ] Ecological, evolutionary & environmental sciences

For a reference copy of the document with all sections, see nature.com/documents/nr-reporting-summary-flat.pdf

Life sciences study design

All studies must disclose on these points even when the disclosure is negative.

Sample size	All available biologic samples from the described cohorts were utilized.
Data exclusions	No data was excluded from these analyses.
Replication	PhIP-Seq was performed on a subset of biological samples in duplicate to ensure reproducibility of individual antibody profiles. All replication attempts were successful
Randomization	Allocation of individuals to dengue challenge groups was random.
Blinding	All experimentation was performed without knowledge of sample groups. Participants in the dengue challenge study were randomized ...

Reporting for specific materials, systems and methods

We require information from authors about some types of materials, experimental systems and methods used in many studies. Here, indicate whether each material, system or method listed is relevant to your study. If you are not sure if a list item applies to your research, read the appropriate section before selecting a response.

Materials & experimental systems

n/a	Involved in the study	
☒	☐	Antibodies
☐	☒	Eukaryotic cell lines
☒	☐	Palaeontology and archaeology
☐	☒	Animals and other organisms
☐	☒	Clinical data
☒	☐	Dual use research of concern
☒	☐	Plants

Methods

n/a	Involved in the study	
☒	☐	ChIP-seq
☒	☐	Flow cytometry
☒	☐	MRI-based neuroimaging

Eukaryotic cell lines

Policy information about cell lines and Sex and Gender in Research

Cell line source(s)	[source of vero cells]
Authentication	Cell lines were not authenticated.
Mycoplasma contamination	Cell lines were not tested for mycoplasma.
Commonly misidentified lines (See ICLAC register)	No commonly misidentified cell lines were used in this study.

Animals and other research organisms

Policy information about studies involving animals; ARRIVE guidelines recommended for reporting animal research, and Sex and Gender in Research

Laboratory animals	Goats age 3 months and adult (Capra hircus), adult Alpacas (Lama pacos), adult Horses (Equus ferus caballus).
Wild animals	No wild animals were used in this study
Reporting on sex	Sex of animals was not considered.
Field-collected samples	This study does not include samples collected from the field.
Ethics oversight	Animal protocols were approved by the Colorado State University Institutional Animal Care and Use Committee

Note that full information on the approval of the study protocol must also be provided in the manuscript.

Clinical data

Policy information about clinical studies

All manuscripts should comply with the ICMJEguidelines for publication of clinical research and a completedCONSORT checklist must be included with all submissions.

Clinical trial registration	dengue challenge trials: NCT02392325, NCT00473135, NCT02317900, and NCT02873260
Study protocol	Full dengue challenge protocols available at ClinicalTrials.gov
Data collection	NCT02392325 occurred from March 2015 through February 2017.<br>NCT00473135 occurred from May 2007 through January 2010.<br>NCT02317900 occurred from December 2014 through November 2016.<br>NCT02873260 occurred from August 2016 through November 2017.<br>Data was collected at:<br>Center for Immunization Research, Johns Hopkins School of Public Health<br>Baltimore, Maryland, United States, 21205<br>and<br>University of Vermont Testing Center<br>Burlington, Vermont, United States, 05405
Outcomes	Dengue neutralization, incidence/intensity of adverse events, and dengue viremia
